# Alexithymia and Emotional Deficits Related to Posttraumatic Stress Disorder: An Investigation of Content and Process Disturbances

**DOI:** 10.1155/2022/7760988

**Published:** 2022-01-22

**Authors:** Ewa A. Ogłodek

**Affiliations:** Department of Health Sciences, Jan Dlugosz University in Czestochowa, Armii Krajowej 13/15 Street, 42-200 Czestochowa, Poland

## Abstract

**Background:**

Posttraumatic stress disorder (PTSD) is a debilitating mental disorder that develops in the aftermath of traumatic life experiences, especially those that occurred in childhood. PTSD is associated with intrusive memories, distressing dreams, dissociative reactions, avoidance of trauma-related stimuli, negative mood and sense of well-being, increased arousal and irritability, and clinically significant distress and impaired functioning. *Case Presentation*. The following case report presents a 42-year-old male displaying symptoms of PTSD, alexithymia, and depression.

**Conclusion:**

Untreated alexithymia may aggravate the trauma and cause the development of PTSD and depression.

## 1. Introduction

Firefighters are exposed to various chemicals and physical hazards and can experience a significant number of accident and injuries during their duty and emergency services [1, 2]. Major physical and chemical perils include extreme heat, toxic compounds, and loud noises [3, 4]. Due to this, firefighters are at high risk of developing posttraumatic stress disorder (PTSD). It is a mental disorder that an individual can develop following a traumatic event. Examples of distressing events involve experiencing, witnessing, or facing an event or events such as death, serious injury, or threat thereof, as well as a threat to the physical integrity of an individual or others [5, 6]. Common reactions to such experiences include intense fear, helplessness, or terror. It is important to note that a given traumatic event is persistently relived in one (or more) of the following ways: recurrent, intrusive, and distressing recollection of the event through images, thoughts, and feelings; recurrent, distressing dreams about the event; a sense of reliving the event, delusions, hallucinations, and dissociative episodes of flashbacks; and severe psychological distress [7, 8]. An important feature of PTSD is persistent avoidance of stimuli associated with the traumatic event. Avoidance may be associated with alexithymia [9–12]. It is a personality trait that reflects deficits in the cognitive processing and regulation of emotions. It may contribute to personality changes after a traumatic experience. Alexithymia may be related to the repeated experience of trauma and the nervous system's susceptibility to stress [13, 14]. Also, it may refer to a preexisting condition that increases the probability of developing PTSD. Multiple traumatization is another factor considered to increase the risk of alexithymia. Both alexithymia and PTSD exhibit emotional numbing, a feature indicative of impaired affect regulation [15, 16].

This report discusses the development of PTSD symptoms in a 42-year-old firefighter who was also diagnosed with alexithymic traits and depressive syndrome. Attention was paid to the chronicity of depressive symptoms in individuals with alexithymia traits following a history of PTSD.

## 2. Case Presentation

A 42-year-old firefighter visited his family physician 2 weeks after surviving a difficult rescue in a high-rise building. The reason for visiting the doctor included headaches, muscle pain especially in the shoulder girdle muscles, and then torticollis. The patient also complained of stress. Having examined the man and received blood test and head magnetic resonance imaging (MRI) results, the primary care physician found no abnormalities. The doctor recommended analgesics—diclofenac 100 mg and paracetamol 500 mg, and sleep-aid hydroxyzine 25 mg. The patient also received a 1-month sick leave from work and was recommended to see a psychiatrist. The patient feared the stigma associated with psychiatric treatment and thus delayed his visit with a psychiatrist.

The man did not see a psychiatrist until seven months after the fire because he continued to experience the symptoms of stress and distress. The psychiatrist carefully took the patient's medical history. It was found that the man was married and had three children (two boys and a girl—aged 7-12 years). The patient had been in the firefighting profession for 5 years, as had his father and grandfather, who were also firefighters. As a child, he was taught that “you should never break down,” “you always have to be strong,” “feeling weak is a failure in life,” and “a real man never breaks down.” The patient wanted to live up to these beliefs in life and pursue a profession as a firefighter to “not let his father and grandfather down.” The man was musically gifted and had once thought about attending a music conservatory. However, he did not pursue that education because his father was very insistent that he goes to firefighter school.

At his first appointment with the psychiatrist, the patient recalled that his father was a very closed-minded person and reluctant to express feelings. He would speak little about difficult situations in his job as a firefighter. The patient also described himself as an introvert and said that he did not like to talk about his experiences and difficulties, preferring to suffer in solitude. The patient was found to exhibit alexithymic traits [17, 18]. Therefore, the Polish version of the Toronto Alexithymia Scale-20 [TAS-20] was used to measure the level of alexithymia [19]. The patient scored 65 on the scale, which confirmed the presence of alexithymia. The patient found it difficult to recognize his emotional states as well as feel, experience, understand, distinguish, and verbalize his own feelings and emotions. Moreover, he was unable to distinguish emotions from physiological changes in the body (e.g., vegetative symptoms) and was prone to negative emotions. The patient focused excessively on somatic sensations, lost the ability to dream and fantasize, and had an excessively operational (concrete) way of thinking. Also, the man reported that he often felt depressed and anxious. Therefore, he would avoid difficult situations, as he considered himself incapable of coping with stress or take advantage of external support.

During subsequent visits with the psychiatrist, the patient complained of intrusive memories of the fire in which he saw children burning. Since experiencing the fire, he had become very sensitive to noise to such an extent that even door closing would irritate him. The patient, after a month of sick leave, began to continue doing his job to distract himself from his experience. He was reluctant to talk to others about what he had been through. The patient began to actively avoid people who initiated conversation about what he had experienced in the rescue. He had a sense of reliving the traumatic event. He experienced intrusive cognitive content (flashbacks), avoided stimuli in his environment that reminded him of the experience, and attempted to avoid memories, thoughts, and feelings associated with the event.

After returning to work, his mental health deteriorated again. He had difficulty sleeping, lost his appetite, began experiencing heart palpitations, and had feelings of discomfort in his stomach, feelings of fear, and a sense that something bad was about to happen (premonition). He was constantly worried, his mood worsened, and he developed the feelings of guilt, wondering why he had survived when so many people had died in the fire. He was afraid of being alone. Also, the patient had difficulty communicating with those around him, including his own family. He also had intrusive images of what might have happened and imagined his children dead. The man had flashbacks of going up to the 10th floor of a high-rise building located in the same neighborhood where the fire had occurred, which happened several times a week while visiting his parents. These flashbacks would make him feel hot and sweaty. He would also wake up with nightmares about the fire. The man would put the memories of the fire out of his mind, especially before bedtime. He would avoid places that he associated with the trauma and the neighborhood where the rescue operation had taken place. In addition, the patient had many symptoms of hyperactivity. He had difficulty sleeping and concentrating, felt irritable, and was hypervigilant.

The man also developed depression 9 months after the fire. He was depressed, tearful, and unmotivated and preferred to stay home rather than engage in his previous activities, such as seeing friends. The patient met the criteria for PTSD and major depression as defined by the Diagnostic and Statistical Manual of Mental Disorders, 5th edition (DSM-V; American Psychiatric Association) [20].

As a result, pharmacologic treatment was initiated with bupropion (modified-release film-coated tablets) 150 mg/d and trazodone 100 mg/d for a period of 1 month. In the second month of therapy, treatment was modified—bupropion was increased to 300 mg/d, and trazodone 150 mg/d was increased as well. Pharmacological treatment at these doses was continued for 4 months. Then, the treatment was modified to bupropion (150 mg/d) and trazodone (50 mg/d) for another 2 months. After 6 months, the medications were discontinued. The patient was found to have achieved improvement in terms of mood and psychomotor drive, anxiety, withdrawal thoughts, apathy, anhedonia, and sleep disorder. However, the patient continued to have catastrophic thinking, hypervigilance, hyperactivity, and flashbacks and continued to experience avoidance of memories, thoughts, and feelings related to the experienced trauma. After psychiatric treatment, the patient decided to undergo psychotherapy.

The patient undertook 12 weeks of Cognitive Processing Therapy (CPT) followed by the second type of Prolonged Exposure (PE) therapy for PTSD. The man learned to recognize the automatic thoughts that sustained his PTSD symptoms. In the next stage of therapy, he learned to use Socratic questions and other techniques designed to challenge unhelpful thoughts so that a shift in thinking could occur. Once the patient had developed the ability to identify and change thoughts (with the help of the therapist), he was prepared to be able to use them independently after the process was complete. In the second type of prolonged exposure therapy, the therapist would discuss with the patient any emotions that would arise in him during the session. The next stage of therapy was in vivo exposure, applied outside the therapist's office. Therapeutic tasks were also performed during breaks between sessions. In the planning of in vivo exposure, a certain gradation of anxiety-provoking stimuli was used, and the patient was gradually engaged in contact with places, situations, and objects that appeared dangerous. In addition, breathing training to regulate excessive arousal also played an important role. After the end of psychotherapy, the patient did not show any symptoms of mental illness.

## 3. Discussion

Based on the available literature, the risk of PTSD among firefighters varies from 6.5% to 50%, which is significantly higher than that in the general population [21]. PTSD manifests as negative thoughts about oneself, other people, or the world; hopelessness about the future; memory problems, e.g., inability to remember important aspects of the traumatic event; difficulty maintaining close relationships; separating oneself from family and friends; lack of interest in activities that once were considered enjoyable; experiencing negative emotions; feeling emotionally numb; being easily startled or frightened; always being on guard for danger; difficulty sleeping and concentrating; and irritability, angry outbursts, aggressive behaviors, or a sense of guilt or shame. Factors that contribute to the risk of PTSD in firefighters include personal characteristics, longer work experience, and exposure to severe traumatic events, among other aspects [22].

The presented case emphasizes the importance of personality background and family environment in the development of PTSD. The patient exhibited traits of alexithymia that made it difficult for him to work in the fire service and likely became a cause for the development of PTSD and subsequently depression. The term alexithymia is used to describe problems related to the cognitive processing of affectional stimulation and thereby deficits in regulating emotions and understanding physiological correlates of emotions. The deficits relate to difficulties in recognizing and verbalizing emotions and refer to thinking which only concentrates on external factors that evoke emotional arousal, i.e., operational style of thinking [23, 24].

Trauma experienced by a child in the form of emotional negligence, or emotional violence, evokes chronic agitation. This requires that such a child needs to develop a strategy to cope with negative emotions [25, 26]. The most frequently applied strategies are disconnection from emotions and failure to communicate experienced feelings to others. The two strategies may contribute to the development of alexithymia. As Schore showed, due to persistent stress in which a child is raised, the inefficient regulation of autonomic nervous system (ANS) is carried out by higher centers in the central nervous system (CNS), which is reflected by the disturbance of central regulation of sympathetic nervous system, as well as the hypothalamus–pituitary gland–adrenal cortex axis (HPA) [27]. It has been shown that the loss of such regulation means that in a stressful situation, the mechanism of combined mutual autonomous control gives way to the mechanism of combined nonmutual autonomous control. This leads to the extremely high arousal of both sympathetic nervous system and parasympathetic nervous system [28]. Traumatic experiences at young age “excite the brain's limbic system,” leaving the permanent physiological responsiveness there and restraining the capability of coping with stressors in the future [29, 30]. The consequence of experiencing permanent distress evoked by traumatic relationships in the early period of life is the progressive decline in the ability to either adjust or undertake defensive actions. This, in turn, enables an affected individual to act in line with one's own interest or register emotions and pain. Studies also show that childhood is a crucial period for mental development, and thus, any neglect experiences have been confirmed as risk factors for a variety of psychological disturbances and personality disorders [31].

Other authors [32, 33] claim that those with childhood emotional neglect experiences are more prone to developing alexithymia. They can contribute to impaired emotional development and disrupt the biological and psychological processes of affect regulation, which may lead to alexithymia. Moreover, children who are emotionally neglected in the family environment often fail to learn to rely on others, even when they need help to regulate their emotional experience or psychological state. Another study has found that childhood trauma experiences can affect people's ability to communicate and identify emotions [34, 35]. Moreover, Young and Widom confirmed that childhood emotional neglect can have a negative impact on children's understanding and recognizing emotions, thereby leading to alexithymia [36].

The patient presented in this case report was found to have difficulty benefiting from psychiatric and psychological visits. The obstacle to seeing a psychiatrist was his mistaken core beliefs like: “one must always be strong,” and “feeling weak is a failure in life.” The concept of anxiety-depressive disorder was interpreted by the patient as an expression of weakness and life failure. This would explain both the patient's fear of seeking psychotherapy and the initial reluctance to seek pharmacotherapy. Clinicians should be aware that this attitude on the part of the patient is quite common and is also related to the prevailing public perception of being negatively marked socially once the patient begins to benefit from psychiatric treatment. Therefore, it is important to reformulate false negative beliefs about psychiatric treatment and psychotherapy during the therapeutic process and build a new conceptual apparatus about mental health. This is to protect the health of people working under chronic stress. There are also reports emphasizing that the formation of appropriate attitudes toward the need for psychiatric treatment contributes to the reduction of PTSD and depression. Thus, in this case, the patient, despite previous negative attitudes toward psychiatric and psychotherapeutic treatment, broke stereotypes and underwent effective therapy [37, 38].

The following case report indicates that both PTSD and depression may be comorbid, leading to possible development of an adverse illness continuum in a patient with chronic stress and alexithymic traits. Adverse childhood experiences can have a tremendous impact on lifelong health and opportunity. The experiences of developmental trauma are chronic, and as the child is not able to avoid them, they can result in permanent disorders at various levels of development: emotional, cognitive, and somatic one. Alexithymia and dissociation may overlap in the process of handling stressful situations, contributing, at the same time, to the increase of deficits in the affective and cognitive sphere of behavior regulation.

## 4. Conclusion

With regard to the high prevalence of PTSD among firefighters and the impact of this condition on life quality, it is crucial to promptly identify individuals at risk of PTSD.

In addition, understanding the health consequences of PTSD can be helpful in implementing mental health prevention among firefighters. Future research should focus on specific types of traumatic experiences to explore their associations with alexithymia in more detail.

## Data Availability

Data is available on request through the author.
